# Therapeutic targeting of CBP/β-catenin signaling reduces cancer stem-like population and synergistically suppresses growth of EBV-positive nasopharyngeal carcinoma cells with cisplatin

**DOI:** 10.1038/srep09979

**Published:** 2015-04-21

**Authors:** King Chi Chan, Lai Sheung Chan, Joseph Chok Yan Ip, Carman Lo, Timothy Tak Chun Yip, Roger Kai Cheong Ngan, Ricky Ngok Shun Wong, Kwok Wai Lo, Wai Tong Ng, Anne Wing Mui Lee, George Sai Wah Tsao, Michael Kahn, Maria Li Lung, Nai Ki Mak

**Affiliations:** 1 Department of Biology, Hong Kong Baptist University, P.R., China; 2 Department of Clinical Oncology, University of Hong Kong, P.R., China; 3 Department of Anatomical and Cellular Pathology and State Key Laboratory in Oncology in South China, The Chinese University of Hong Kong, P.R., China; 4 Department of Clinical Oncology, Queen Elizabeth Hospital Hong Kong, P.R., China; 5 Center for Nasopharyngeal Carcinoma Research, University of Hong Kong, P.R., China; 6 Clinical Oncology, Pamela Youde Nethersole Eastern Hospital, P.R., China; 7 Department of Anatomy, University of Hong Kong, P.R., China; 8 Department of Biochemistry and Molecular Biology, Norris Comprehensive Cancer Center, Keck School of Medicine, University of Southern California, Los Angeles, CA, USA

## Abstract

Nasopharyngeal carcinoma (NPC) is an EBV-associated epithelial malignancy prevalent in southern China. Presence of treatment-resistant cancer stem cells (CSC) may associate with tumor relapse and metastasis in NPC. ICG-001 is a specific CBP/β-catenin antagonist that can block CBP/β-catenin-mediated transcription of stem cell associated genes and enhance p300/β-catenin-mediated transcription, thereby reducing the CSC-like population via forced differentiation. In this study, we aimed to evaluate the effect of ICG-001 on the CSC-like population, and the combination effect of ICG-001 with cisplatin in the C666-1 EBV-positive NPC cells. Results showed that ICG-001 inhibited C666-1 cell growth and reduced expression of CSC-associated proteins with altered expression of epithelial-mesenchymal transition (EMT) markers. ICG-001 also inhibited C666-1 tumor sphere formation, accompanied with reduced SOX2^hi^/CD44^hi^ CSC-like population. ICG-001 was also found to restore the expression of a tumor suppressive microRNA-145 (miR-145). Ectopic expression of miR-145 effectively repressed SOX2 protein expression and inhibited tumor sphere formation. Combination of ICG-001 with cisplatin synergistically suppressed *in vitro* growth of C666-1 cells and significantly suppressed growth of NPC xenografts. These results suggested that therapeutically targeting of the CBP/β-catenin signaling pathway with ICG-001 can effectively reduce the CSC-like population and combination with cisplatin can effectively suppress the growth of NPC.

Nasopharyngeal carcinoma (NPC) is an epithelial malignancy arising from the nasopharynx. It has a relatively high prevalence in southern China with an annual incidence of >20 per 100,000^1^. NPC is consistently associated with Epstein-Barr virus (EBV) latent infection. The EBV-encoded genes and cellular microRNAs (miRNAs) are believed to be involved in the pathogenesis of NPC^2^. C666-1 is the only EBV-positive NPC cell line carrying native EBV genome and is preferred as a suitable model for translational study of EBV-associated NPC^3^. Currently, the standard treatment for NPC is radiotherapy or combined chemo-radiotherapy with cisplatin-based regimens^4^. Although primary NPC can be successfully treated with radiotherapy and chemo-radiotherapy, the treatment outcomes for locally advanced and metastatic NPC remains unsatisfactory. High rates of local relapse and distant metastasis are the major concern for treatment failure^5^. Accumulating evidence suggested that a subpopulation of cancer cells with self-renewal and stem-like cell properties, namely cancer stem cells (CSCs), play an important role in tumor initiation and resistance to radio- and chemotherapy^6^,^7^. The existence of CSCs may explain the high rate of tumor relapse after standard therapies^8^. Therapeutic targeting of the CSC population is a novel strategy to overcome therapeutic resistance and tumor relapse after cancer treatment^9^,^10^.

Wnt signaling is one of the key CSC self-renewal signaling pathways^11^. Multiple stem cell-related genes such as CD44, survivin, and c-myc are Wnt-regulated genes. Early studies on the gene expression profile of NPC showed that the expression of a number of Wnt signaling components, including wingless-type MMTV integration site family, member 5A (Wnt5A), Frizzled homolog 7 (FZD7), β-catenin, and CREB-binding protein (CBP) are frequently activated, and the aberrant increased expression of β-catenin is associated with poor prognosis in NPC^12^,^13^,^14^. In addition to the overexpression of the Wnt components, epigenetic inactivation of tumor suppressor Wnt inhibitory factor-1 (WIF-1) was also found to be involved in the activation of the Wnt pathway and the metastasis of NPC^15^,^16^. In NPC, the Wnt signaling pathway was aberrantly activated and the expression of the Wnt-regulated stem cell associated genes was also found to be up-regulated^17^. To achieve a better translational outcome, therapeutic targeting of the Wnt signaling in NPC may reduce the CSC population and, therefore, sensitize the cells to conventional treatment.

Small molecules targeting the Wnt signaling pathway have recently been introduced to pre-clinical and clinical studies^18^,^19^. Kahn and co-workers have previously found that ICG-001, a small molecule Wnt modulator, can safely eradicate the drug-resistant CSC-like population and initiate cell differentiation in leukemia and solid cancer models^20^,^21^,^22^. In the canonical Wnt pathway, nuclear β-catenin binds to the coactivator CBP to mediate transcription of genes associated with stem cell proliferation and cell pluripotency. ICG-001 binds specifically to CBP and acts as a CBP/β-catenin antagonist, which blocks transcription of genes associated with stem cell proliferation and self-renewal, thereby reducing the stem cell population. This also facilitates the interaction of β-catenin with the highly homologous coactivator p300 to initiate transcription of genes associated with cell differentiation^23^,^24^. As Wnt signaling is deregulated in NPC, we hypothesized that pharmacological intervention of Wnt signaling with the CBP/β-catenin antagonist ICG-001 could be used to reduce the CSC-like population. Here, we studied the effect of ICG-001 on growth of the EBV-positive C666-1 CSC-like population through 3-D tumor sphere formation assay. We also found that the growth inhibitory effect of ICG-001 is correlated with the downregulated expression of the NPC CSC-like markers SOX2 and CD44, and the upregulated expression of the tumor suppressive microRNA-145 (miR-145). Combination of ICG-001 with the conventional drug cisplatin showed a synergistic growth inhibitory effect on the C666-1 cell growth and significant tumor suppressive effect on C666-1 and xeno-2117 EBV-positive NPC xenograft models. This study provides evidence for use of CBP/β-Catenin antagonists (i.e. ICG-001 or PRI-724) as potential CSC-targeting drugs and the combination effect of ICG-001 with conventional therapy in the improvement of treatment outcome in NPC.

## Results

### Growth inhibitory effect of ICG-001 on C666-1 EBV-positive NPC cells

**Figure 1:**
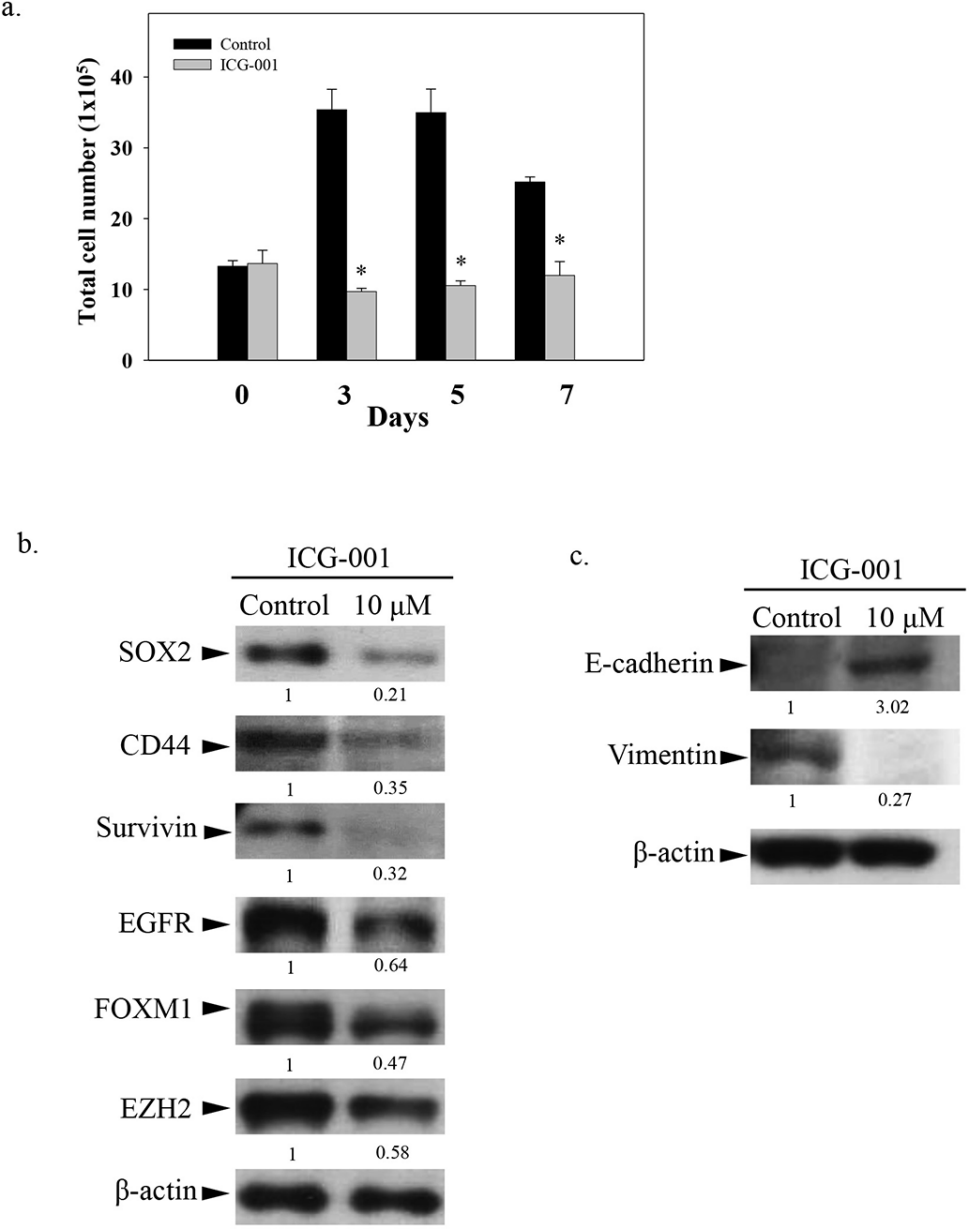
Effect of ICG-001 on the growth of EBV-positive C666-1 cells. (a) Cell growth assay. Total number of viable cells was counted on days 3, 5, and 7 after ICG-001 treatment. Results were expressed as the mean ± S.D. of three experiments; *p < 0.05. (b) Western blotting analysis of SOX2, CD44, Survivin, EGFR, FOXM1, and EZH2 expression on day-7. (c) Western blotting analysis of epithelial-mesenchymal transition (EMT) markers, E-cadherin and vimentin on day-7. The gels have been run under the same experimental conditions with different percentage of gels. The full-length blots were presented in the supplementary Figure S1.

The cell growth assay was initially used to evaluate the effects of ICG-001 on C666-1 cells. Figure 1a showed the number of untreated control C666-1 cells reaching a maximum plateau level at 3 to 5 days after incubation. However, the growth of C666-1 was significantly inhibited by 10 µM ICG-001 (*p<0.05). We next studied the effect of ICG-001 on the expression of cell growth and CSC associated proteins. In Figure 1b, Western blotting analysis showed that ICG-001 downregulated the expression of SOX2, CD44, survivin, EGFR, FOXM1, and EZH2 in C666-1 after 7 days of treatment. The growth inhibitory effects of ICG-001 clearly correlated with the downregulated expression of a group of proteins associated with the development of CSCs in C666-1. Epithelial-mesenchymal transition (EMT) has been implicated in the transition process of CSC formation^25^. Next, we studied the expression of EMT markers in ICG-001-treated C666-1 cells. Western blot analysis showed the re-expression of E-cadherin (epithelial marker) and decreased expression of vimentin (mesenchymal marker) after 7 days of ICG-001 treatment (Figure 1c). This result indicates that ICG-001 decreases the mesenchymal stem-like phenotype and increases the differentiated epithelial status of the NPC cells.

### ICG-001 inhibits the growth of tumor spheres

**Figure 2:**
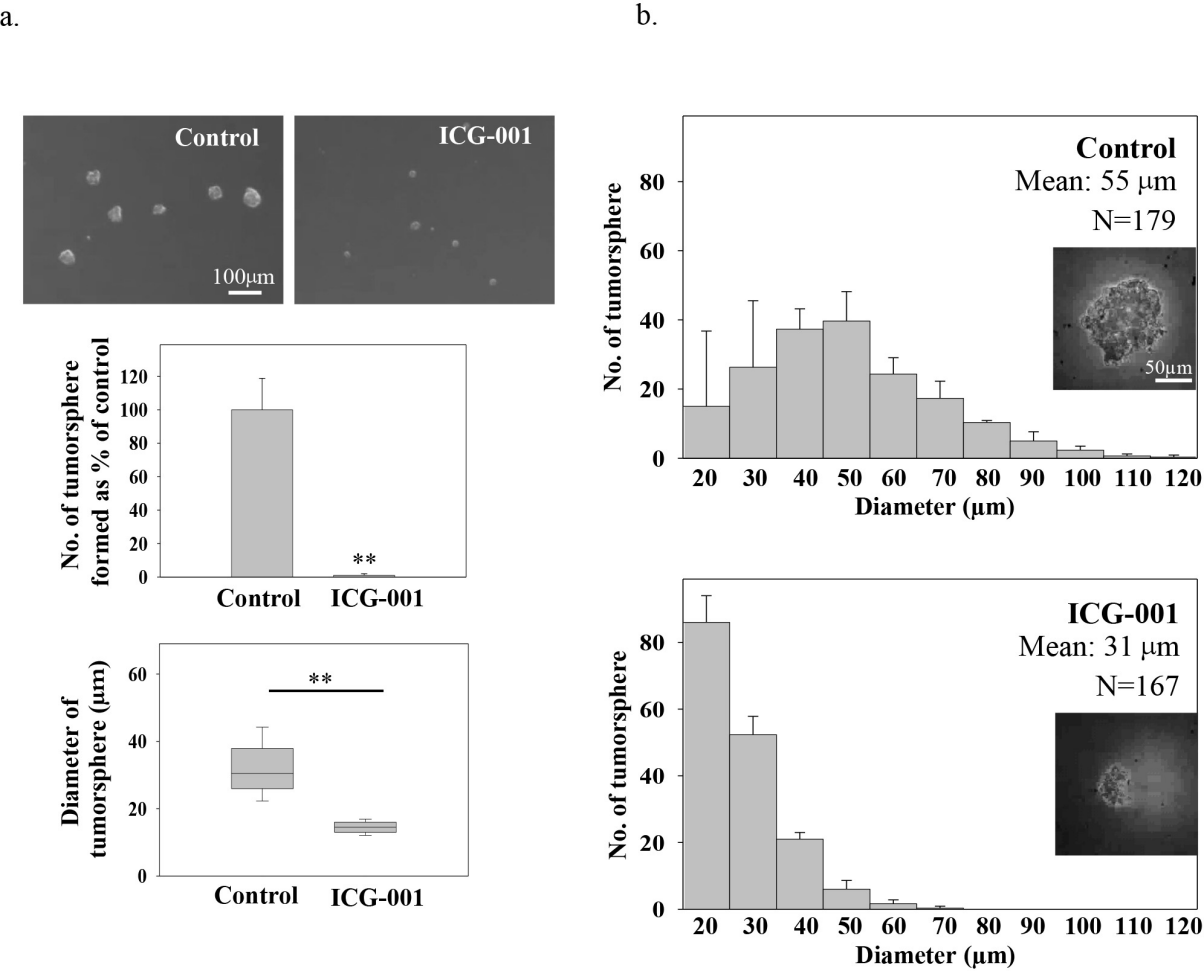
Effect of ICG-001 on the growth of C666-1 in the 3-D tumor sphere formation and tumor sphere growth assay. (a) Effect on the tumor sphere formation. ICG-001 was added on day 0, when plating the cell into ultra-low attachment plate. The number and size of tumor sphere formed from C666-1 with and without ICG-001 treatment were compared. (b) Effect on the growth of established spheroids. Tumor spheres were allowed to form for 7 days, then the established tumor spheres were incubated with ICG-001 for another 7 days. The number and diameter of tumor spheres measured were presented as tumor sphere size profiles. Results were expressed as the mean ± S.D. of three experiments; **p < 0.02.

Next, the tumor sphere formation assay was employed to study the effect of ICG-001 on the growth of CSC-like cells. The tumor sphere formation assay is frequently used as an *in vitro* assay to enrich CSC-like cells under the 3-D culturing condition for functional studies^26^. In this study, C666-1 cells were incubated with ICG-001 for 7 days and the number and diameter of tumor spheres were quantified. Results in Figure 2a showed that ICG-001 significantly (**p<0.02) inhibited the formation of tumor spheres. A clear inhibition of both number and size of tumor sphere formation were found after the treatment. To further study the effect of ICG-001 on the growth of established tumor spheres, C666-1 cells were allowed to grow and form tumor spheres without any treatment for 7 days. The established tumor spheres were then incubated with ICG-001 for another 7 days. Both the number and diameter of tumor spheres were measured and presented as a tumor sphere size profile. Results in Figure 2b showed that the majority of the ICG-001-treated tumor spheres remained small, with a mean diameter of 31 µm, while the majority of the untreated tumor spheres continued to grow as the size profile shifted to larger diameters, with a mean diameter of 55 µm. This observation indicated that ICG-001 effectively inhibited the further expansion of tumor spheres in the 3-D culture.

### ICG-001 reduced SOX2 and CD44 expression in C666-1 tumor spheres

**Figure 3:**
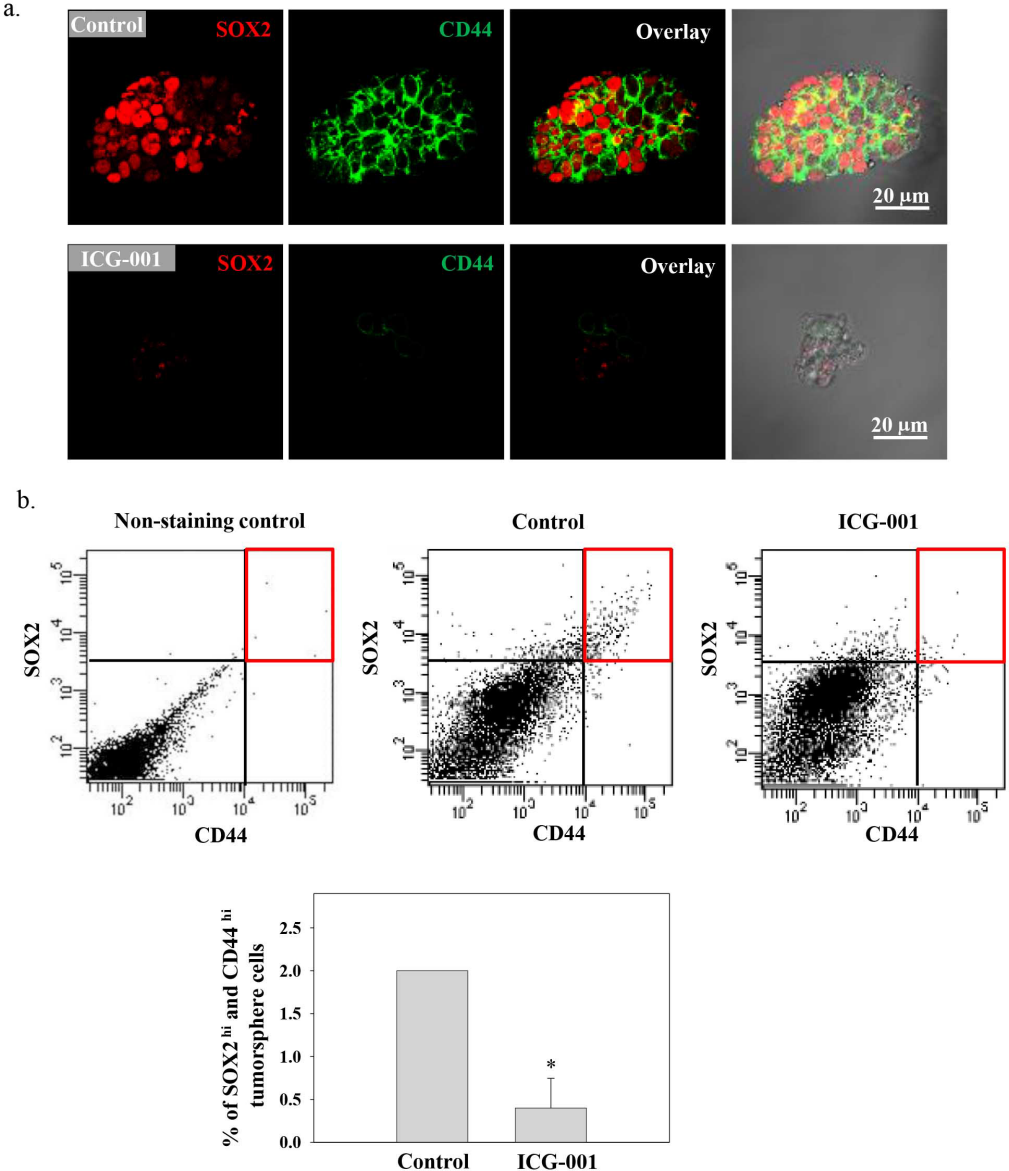
ICG-001 reduced SOX2 and CD44 expression in C666-1 tumor sphere. (a) Confocal staining showing pseudocolor red for SOX2 and green for CD44. Loss of both SOX2 and CD44 expression was observed in ICG-001-treated C666-1 tumor sphere. (b) FACS analysis of spheroid cells stained with Alex Fluor® 647 conjugated SOX2 and Alexa Fluor® 488 conjugated CD44 and gated against the non-stained tumor sphere cells. The SOX2^hi^/CD44^hi^ population was significantly decreased after ICG-001 treatment. Results were expressed as the mean ± S.D. of three experiments; *p < 0.05.

In NPC, both SOX2 and CD44 have been shown to be enriched in the sphere-forming CSC-like populations^27^. After C666-1 tumor sphere growth analysis, the expression of SOX2 and CD44 by the spheroid cells was analyzed using confocal microscopy. A mild to strong immunoreactivity of SOX2 (red) staining in the nucleus and CD44 (green) staining in the membrane was observed in the spheroid cells obtained from the control culture (Figure 3a). The immunoreactivity of SOX2 and CD44 was greatly reduced in the spheroid cells after the treatment of ICG-001. Quantitative expression of SOX2^hi^ and CD44^hi^ population of the spheroid cells was also determined by Fluorescence-activated Cell Sorting (FACS) analysis. Figure 3b shows the dot-plot of SOX2^hi^/CD44^hi^ population. The results were gated against the non-stained tumor sphere cells and quantified in the bar chart. There was a 5-fold reduction of the SOX2^hi^/CD44^hi^ population after ICG-001 treatment (*p<0.05).

### Involvement of SOX2 in the growth of C666-1 tumor sphere formation

**Figure 4:**
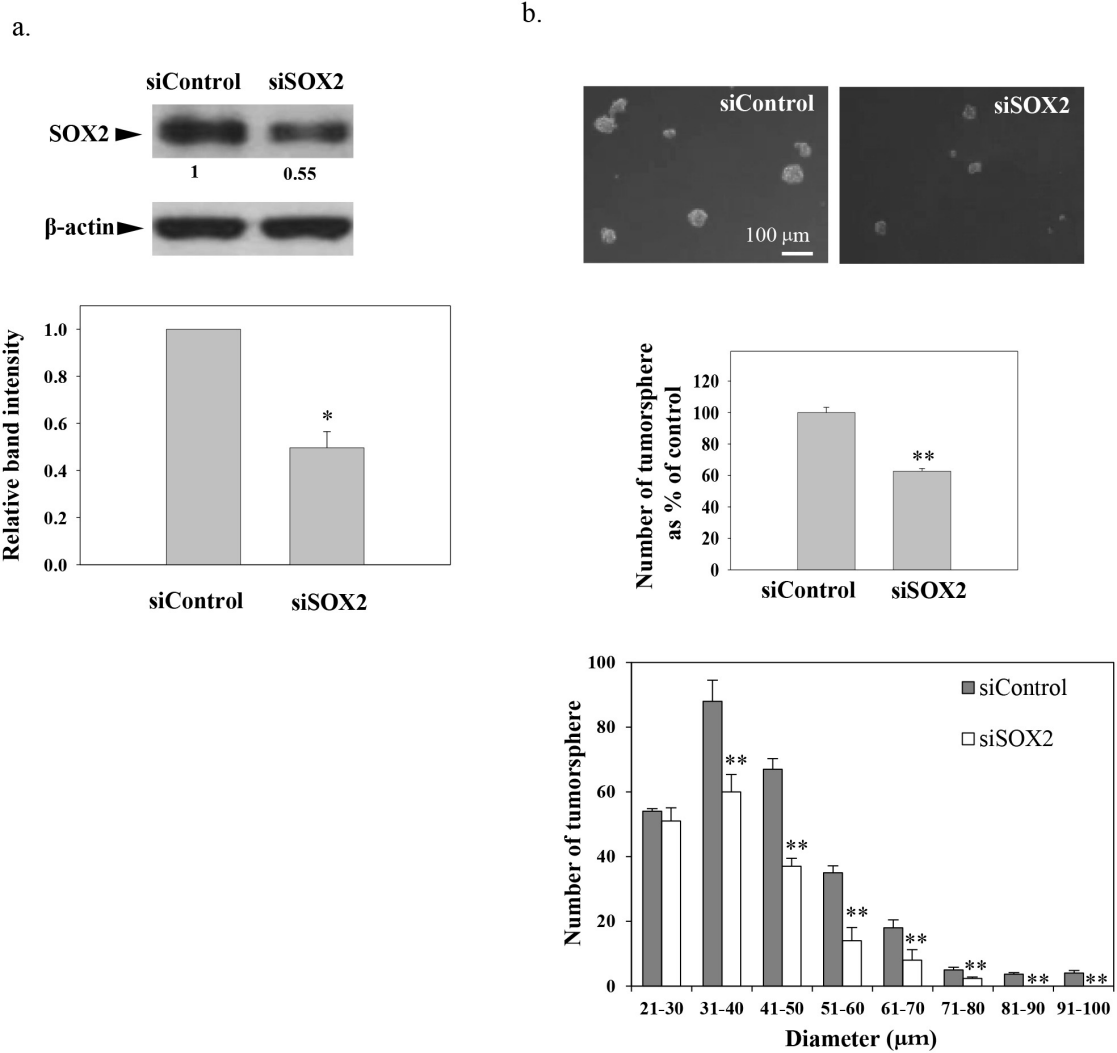
The role of SOX2 in tumor sphere formation. Transfection of C666-1 with SOX2 siRNA led to (a) decreased SOX2 expression and (b) decreased number and size of tumor sphere formation. Results were expressed as the mean ± S.D. of three experiments; *p< 0.05; **p < 0.02. The gels have been run under the same experimental conditions. The full-length blots are presented in the supplementary Figure S2.

The involvement of CD44 in tumor sphere formation has been well-characterized in EBV-positive NPC cells^27^, while the involvement of SOX2 in tumor sphere formation has not been studied in EBV-positive NPC cells. SOX2 is one of the major transcription factors involved in the maintenance of the stem cell pluripotency^28^. Overexpression of SOX2 has been implicated in the regulation of mammosphere formation in the breast cancer model^29^. In the present study, we determined the role of SOX2 in C666-1 tumor sphere formation by using SOX2 siRNA. Figure 4a showed that the expression of SOX2 was reduced in C666-1 after transfection with SOX2 siRNA. After knockdown of SOX2 expression, Figure 4b shows both the number and the diameter of the tumor spheres were significantly reduced in the tumor sphere formation assay (**p<0.02). This finding indicated that SOX2 is involved in the regulation of growth of the sphere.

### ICG-001 restored miR-145 expression, which repressed SOX2 and inhibited tumor sphere formation in C666-1

**Figure 5:**
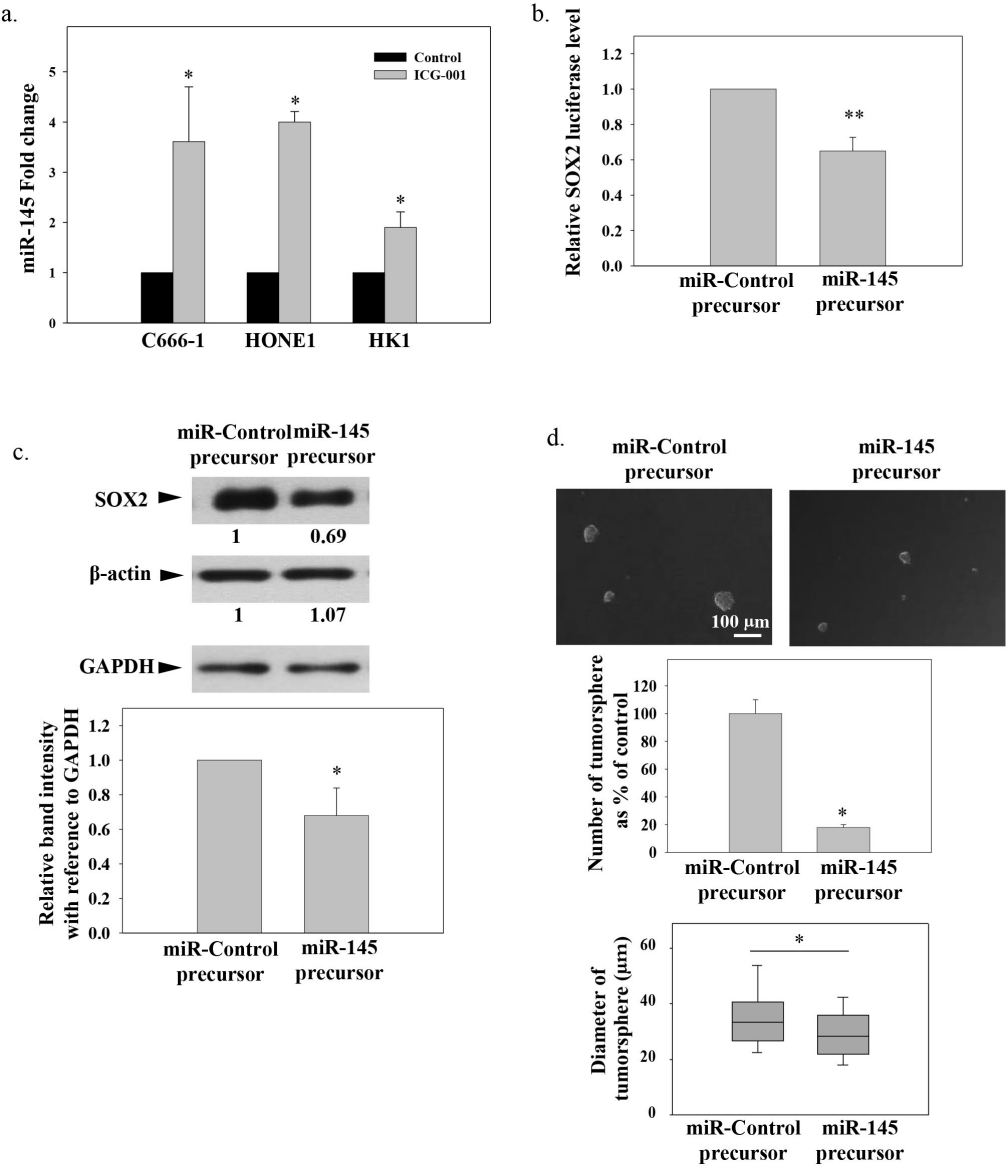
Restoration of miR-145 by ICG-001 and the role of miR-145 in suppression of SOX2 and tumor sphere formation. (a) Expression of miR-145 in ICG-001-treated EBV-positive and -negative NPC cell lines. (b) SOX2 3'UTR Luciferase reporter assay. (c) and (d) Transfection of miR-145 precursor suppressed SOX2 protein expression and reduced number and size of tumor sphere formed in ultralow attachment plate. Results were expressed as the mean ± S.D. of three experiments; *p < 0.05; **p < 0.02. The gels have been run under the same experimental conditions. The full-length blots are presented in the supplementary Figure S3.

Recent study suggested the enrichment of SOX2 expression in tumor spheres may be due to loss of the SOX2 repressing miRNAs^27^. Therefore, we studied the expression of SOX2 repressing miRNAs in NPC cells after ICG-001 treatment. MiR-145 is a known SOX2-repressing miRNA downregulated in NPC tissue^30^,^31^. Realtime PCR results in Figure 5a show that ICG-001 could significantly restore miR-145 expression in both the EBV-positive C666-1 and EBV-negative HONE1 and HK-1 NPC cell lines (*p<0.05). Next, a SOX2 3'UTR luciferase reporter assay was used to further validate the suppressive action of miR-145 on SOX2 expression in C666-1. Figure 5b shows that cotransfection of miR-145 precursor and SOX2 3'UTR reporter effectively reduced luciferase activities compared to miR-precursor control (**p<0.02). Results in Figures 5c and 5d demonstrate that transfection of miR-145 precursor resulted in the downregulated expression of the SOX2 protein in C666-1 (*p<0.05). The number and size of C666-1 tumor sphere formation were also significantly suppressed (*p<0.05). Taken together, ICG-001 was found to inhibit the CSC-like population through restoration of miR-145, which directly binds to SOX2 3'UTR and represses SOX2 expression in C666-1 NPC cell line.

### Synergistic growth inhibitory effect of ICG-001 and cisplatin on C666-1 cells

**Figure 6:**
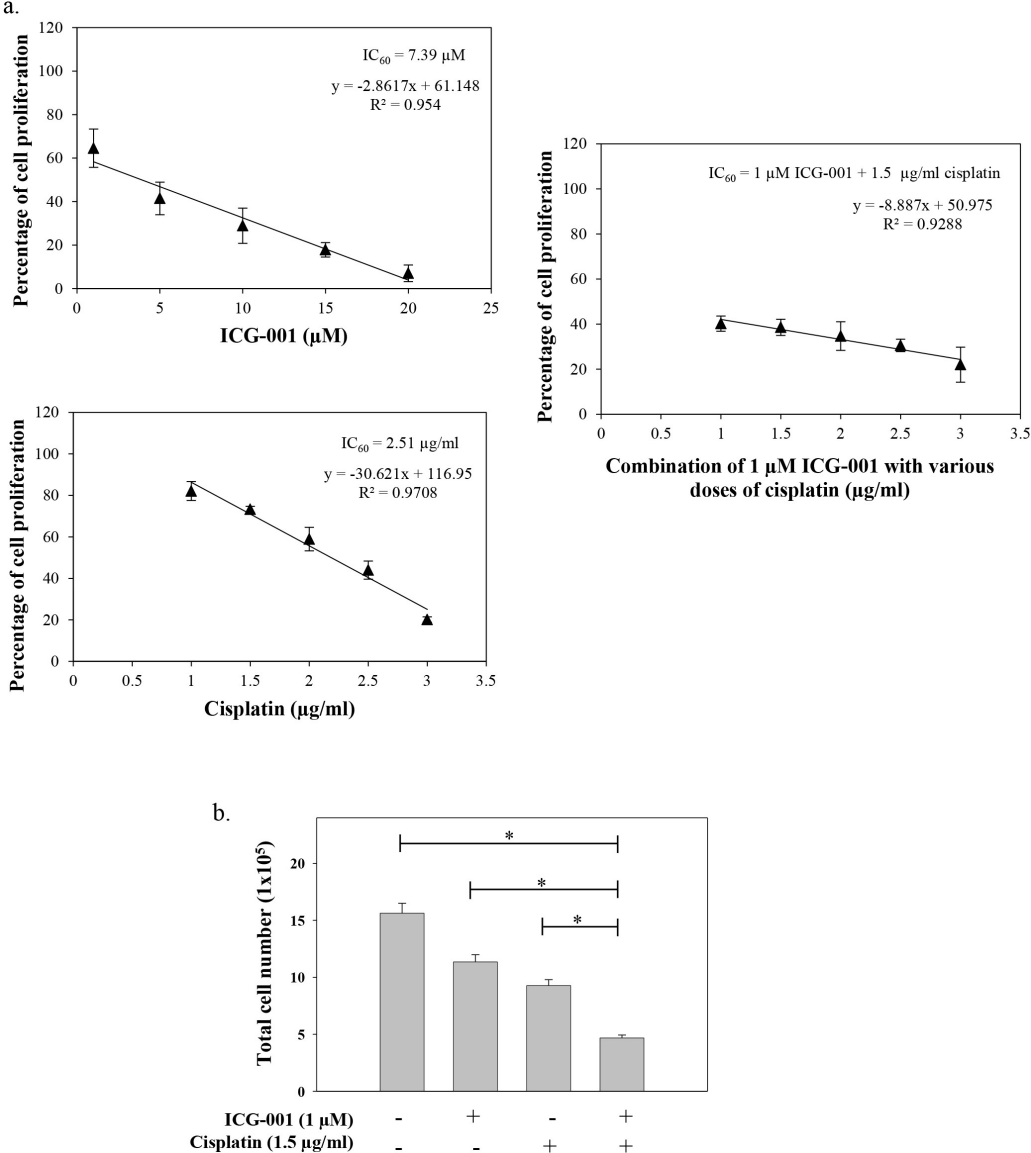
Combination of ICG-001 and cisplatin showed a synergistic growth inhibitory effect on C666-1 cells. (a) MTT assay showing the percentage of cell proliferation after 7 days treatment with various concentrations of ICG-001 and cisplatin. When comparing the IC_60_, the calculated CI value is 0.7, indicating a synergistic growth inhibitory effect of ICG-001 and cisplatin. (b) Cell growth assay showing the total number of proliferating cells counted on day-7 after ICG-001 and cisplatin drug treatment. Combination of ICG-001 and cisplatin showed a greater significant growth inhibitory effect in C666-1 than either drug treatment alone. Results are expressed as the mean ± S.D. of three experiments; *p < 0.05.

The existence of chemo-resistant CSC-like populations within the bulk tumor is believed to be associated with tumor relapse and metastasis after standard treatment procedures^8^. On the other hand, treatment-sensitive bulk tumor cells in the tumor microenvironment may revert back to cells with CSC-like properties through the process of EMT. Hence, elimination of both the CSC-like population and the bulk tumor cells with combination of a novel CSC targeting drug and conventional chemotherapy is one current therapeutic strategy to improve treatment outcome. In this study, we evaluated the combination effect of ICG-001 with cisplatin on the growth of C666-1 cells. In the MTT assay (Figure 6a), various concentrations of ICG-001 and cisplatin were studied as a single agent or in combination on C666-1 cells. The IC_60_ (inhibition of 60% of cell proliferation) of ICG-001 alone was 7.39 µM and of cisplatin alone was 2.51 µg/ml. In the combination of ICG-001 with cisplatin, the IC_60 _of ICG-001 and cisplatin was 1 µM and 1.5 µg/ml, respectively. The calculated combination index (CI) was 0.7, indicating the synergistic inhibitory effect of ICG-001 and cisplatin on C666-1. The combined growth inhibitory effect of 1 µM ICG-001 with 1.5 µg/ml cisplatin was further confirmed by counting the number of viable cells in the cell culture (Figure 6b). Combined treatment of ICG-001 with cisplatin exhibited a greater significant growth inhibitory effect in C666-1 than either drug treatment alone (*p<0.05).

### Combination of ICG-001 and cisplatin significantly suppressed tumor growth in EBV-positive NPC xenografts

**Figure 7:**
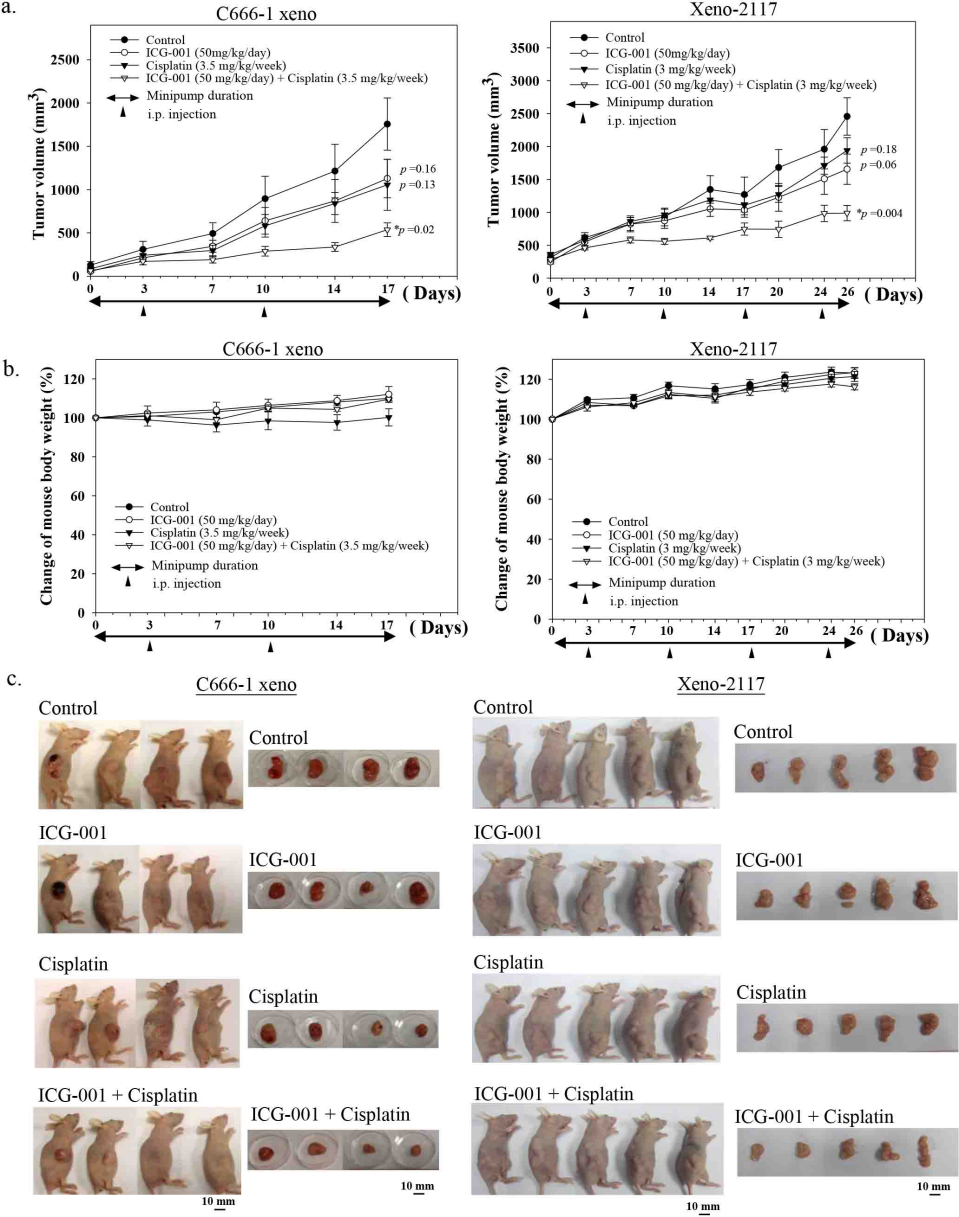
Combination of ICG-001 and cisplatin significantly suppressed tumor growth in EBV-positive NPC xenografts. (a) C666-1 cells and xeno-2117 were subcutaneously injected into the right flanks of nude mice, respectively (n = 4 for C666-1 xenograft and n = 5 for xeno-2117). Drug treatments were started when the tumors became palpable, and the size of tumors was measured twice weekly and plotted as the average tumor volumes versus number of days post-treatment. (b) Percentage change of mouse body weight measured throughout the experiment. (c) Photos of tumor-bearing mice and tumors dissected from mice at the end of experiment.

Next, we studied the combined effect of ICG-001 with cisplatin in the C666-1 and xeno-2117 xenograft models. C666-1 cells and xeno-2117 were subcutaneously inoculated into the right flanks of nude mice, respectively. When the xenografts became palpable, 50 mg/kg/day ICG-001 was delivered via a subcutaneous osmotic minipump implanted into the left flanks of the mice. A previously determined sub-optimal dose of cisplatin (3.5 mg/kg/week for C666-1 xenografts and 3 mg/kg/week for xeno-2117 xenografts) was delivered once per week via i.p. injection. PBS delivered via the minipump was used as the ICG-001 vehicle control. 3.3% DMF in saline delivered by i.p. injection once per week was used as the cisplatin vehicle control. The tumor volumes were measured twice weekly and the results were plotted as the average tumor volume versus number of days post-treatment. Results in Figure 7a showed the average tumor volume of C666-1 and xeno-2117 xenografts. For both of the xenograft models, the vehicle control group showed the largest average tumor volume at the end of experiment. Slight tumor suppression was observed in the ICG-001 alone group (C666-1 xenograft, *p* = 0.16; xeno-2117, *p* = 0.06) and cisplatin alone treatment groups (C666-1 xenograft, *p* = 0.13; xeno-2117, *p* = 0.18). A significant tumor suppression was observed in the combination of ICG-001 and cisplatin treatment group (C666-1 xenograft, *p* = 0.02; xeno-2117, *p* = 0.004). The weight of the mice was also measured twice weekly and the change of mouse body weight was plotted in 7b. For C666-1 xenografts, the vehicle control group, ICG-001 alone treatment group and combination treatment group showed a steady increase in the body weight, while cisplatin alone treatment group did not show any gain of body weight during the treatment period. For xeno-2117, a steady increase of mouse body weight was observed in the vehicle control group, ICG-001-alone, cisplatin-alone, and combined treatment group. Combination of ICG-001 and cisplatin significantly suppressed tumor growth in C666-1 and xeno-2117 xenografts without reduction of mouse body weight as compared to the vehicle control mice. Figures 7c shows the images of tumor-bearing mice and tumors dissected from mice at the end of experiment.

## Discussion

Cancer stem cells (CSCs) represent a subpopulation of cells within the bulk tumor that have the capability to undergo self-renewal, drive the tumor growth, and are resistant to radiation and conventional drugs^6^,^7^,^8^. The residual CSCs after conventional treatment can lead to tumor relapse in local and distant regions. Therapeutic targeting of the CSC population may overcome the local relapse and distant metastatic treatment failure in NPC. The existence of CSCs has recently been identified in various types of solid tumors such as brain tumor, breast cancer, ovarian cancer, and NPC^32^,^33^,^34^,^35^. In NPC, both SOX2 and CD44 expression were found to be enriched in the CSC population and serve as potential markers for the CSC population in NPC^27^. In the present study, we demonstrated that the ICG-001 can reduce the growth of tumor spheres and inhibit the production of the SOX2^hi^/CD44^hi^ CSC-like population in EBV-positive C666-1 NPC cells. Compared with other tumors, C666-1 is relatively resistant to cisplatin^36^. Here, we demonstrated that the combination of ICG-001 with cisplatin can effectively suppress tumor growth in the C666-1 and xeno-2117 xenograft models. These results strongly suggest that combination of ICG-001 with cisplatin can enhance the tumor suppressive effect in NPC cells.

Wnt, Notch, and Hedgehog are the three major signaling pathways regulating CSC growth and pluripotency^10^. These signaling pathways may integrate and funnel down into a simple decision point for CSCs to retain potency or initiate differentiation^18^. The binding of nuclear β-catenin with its transcriptional coactivator CBP is an important cell fate determination point for cells to maintain pluripotency or initiate differentiation^18^,^23^. By targeting this final cell fate decision point using CBP/ β-catenin antagonists e.g. ICG-001, we may alter the expression of multiple CSC-associated proteins from different signaling pathways. CSC-associated proteins such as SOX2, CD44, survivin, EGFR, FOXM1, and EZH2 have been shown to play important roles in cell proliferation and maintenance of stemness phenotypes^28^,^37^,^38^,^39^,^40^,^41^. SOX2 is one of the major stem cell pluripotency transcription factors, essential for pluripotency in embryonic stem cells^28^. In NPC, SOX2 has been shown to be expressed in over 90% of tumor tissue and the expression is significantly associated with a poorer distant metastasis-free survival^42^. CD44 is membrane receptor for hyaluronic acid involved in a wide range of cellular functions^37^. Both SOX2 and CD44 are found to be enriched in the NPC CSC population with higher clonal and sphere forming abilities^27^. Survivin is a direct Wnt targeted gene responsible for cell proliferation and anti-apoptosis^38^. The decreased expression of survivin is currently used as an on-target drug effect biomarker in the clinical study of the second generation CBP/β-catenin antagonist PRI-724^18^. EGFR is a tyrosine kinase receptor associated with NPC poor prognosis, cell proliferation, migration, and drug resistance^39^. Therapeutic targeting of EGFR in NPC using cetuximab and gefitinib are currently under clinical evaluations, however, the results are still not promising^43^,^44^. FOXM1 is a member of Forkhead box transcription factor family involved in self-renewal and stem cell proliferation^40^. EZH2 is a component of the polycomb repressive complex 2 that is essential in regulating embryonic development, pluripotency and self-renewal^41^. Both FOXM1 and EZH2 have been implicated as potential therapeutic targets for NPC and are the downstream targets of EBV-activated Hedgehog signaling pathway^45^,^46^,^47^. In the present study, ICG-001 was found to downregulate the expression of these multiple essential CSC-associated proteins and effectively reduce the CSC population.

Tumor sphere formation is a functional assay to enrich the CSC-like cells under serum-free and ultralow attachment conditions. This *in vitro* method closely mimics the *in vivo* tumor growth situation^26^. In the present study, we demonstrated that ICG-001 can reduce tumor sphere formation with concomitant reduction of both SOX2 and CD44 expression. The siRNA knockdown of SOX2 in C666-1 significantly inhibited tumor sphere formation, indicating the involvement of SOX2 in regulating the growth of the CSC-like population in NPC. In a recent NPC CSC study, Lun *et al.* suggested that the enrichment of SOX2 expression in tumor spheres may be due to the loss of SOX2 repressing miRNAs^27^. miRNAs are short noncoding RNAs that regulate gene expression post-transcriptionally and have been recently been reported to regulate the expression of stem cell maintenance factors^48^. In NPC, the expression of miR-145 has been shown to be downregulated^31^ and miR-145 has also been shown to be a SOX2 repressing miRNA in human embryonic stem cells^30^. To further study the underlying mechanism of ICG-001 in the regulation of SOX2 expression, we examined the expression of miR-145 after ICG-001 treatment. ICG-001 restored the expression of miR-145 in both the EBV-positive and EBV-negative NPC cell lines. Transfection of miR-145 precursor into C666-1 significantly repressed SOX2 protein expression and tumor sphere formation. This observation is in line with the previous studies that miR-145 can directly repress SOX2 protein expression, thereby reducing the stem cell phenotype^30^,^49^. According to the miRNA target prediction algorithms, there was a recent concern on the potential influence of miR-145 on the protein expression of β-actin^50^, and, hence, affecting the normalization of the SOX2 protein expression. In the present study, the housekeeping protein GAPDH was used as an internal control in the estimation of SOX2 expression, and, in fact, we did not observe the repressive effect of miR-145 on the expression level of β-actin.

Today, the standard treatment for NPC is radiotherapy or combined chemo-radiotherapy mainly with cisplatin-based regimens^4^. Patients often suffer from the toxic side effects of cisplatin and a significant rate of chemoresistance contributes to NPC treatment failure^51^. Since the CSC-like populations within the bulk tumor are more resistant to conventional therapy^8^, there is a need to identify drugs that can effectively target the CSC-like populations. Combination of CSC targeting drugs with conventional therapy offers a promising therapeutic strategy to overcome treatment failure in NPC. In this study, we first demonstrated ICG-001 can effectively reduce the CSC-like population in C666-1. Next, we showed the combined tumor suppressive effect of ICG-001 with cisplatin in C666-1. C666-1 was previously reported as a cisplatin-resistant cell line^27^. Using a nude mouse tumorigenicity assay, we demonstrated that cisplatin alone did not significantly suppress C666-1 xenograft growth (*p* = 0.13). However, the combination of ICG-001 with cisplatin significantly suppressed the growth of C666-1 xenografts (*p* = 0.02). Also, the overall health condition of the ICG-001 treated mice and importantly the combined treatment mice was better than the cisplatin-alone treated mice. This data provided functional evidence that the combination of ICG-001 with cisplatin could enhance the tumor suppressive effect in NPC xenograft while at the same time ameliorating some of the cisplatin toxicity. The combination of ICG-001 with cisplatin is, therefore, safer and more efficacious for the treatment of NPC tumors than cisplatin alone. PRI-724, a second generation CBP/β-catenin antagonist, approximately 20 times more potent than ICG-001 is currently being used in multiple clinical studies. Phase Ia clinical studies of PRI-724 in advanced solid tumors showed a very acceptable toxicity profile up to 905 mg/m^2^ for 7 day i.v infusion to patients as reported at 2013 American Society of Clinical Oncology (ASCO). A phase Ib trial of PRI-724 combined with gemcitabine for refractory pancreatic cancer , a phase Ib trial of PRI-724 combined with Folfox6 for refractory colorectal cancer, and a phase I/II trial for leukemia are currently under evaluation^18^. Both the pre-clinical and clinical evidence supports the safety and efficacy of CBP/β-catenin antagonists in combination with conventional drug treatment in cancer. Future clinical investigations of PRI724 combined with cisplatin may benefit NPC patients providing a better treatment outcome.

## Conclusion

The presence of cancer stem cells is believed to be associated with tumor relapse and metastasis after NPC standard treatments. Our results suggest that ICG-001 can inhibit the growth of the CSC-like population in EBV-positive NPC cells. ICG-001 can significantly reduce the growth of tumor spheres and the population of spheroid cells with a SOX2^hi^/CD44^hi^ phenotype. Furthermore, the combination of ICG-001 with cisplatin demonstrated enhanced tumor suppressive effects on the EBV-positive NPC cells and xenografts, which may warrant further clinical study in NPC patients.

## Methods

### Chemical and antibodies

Primary antibodies SOX2, CD44, survivin, EGFR, FOXM1, and EZH2 for Western blotting analysis were purchased from Cell Signaling (Danvers, MA). Primary antibody for E-cadherin was purchased from Invitrogen (Carlsbad, CA). Primary antibodies for vimentin and GAPDH were purchased from Santa Cruz Biotechnology (Santa Cruz, CA), and β-actin was purchased from Sigma-Aldrich (St. Louis, MO). Antibodies for immunofluorescence staining Alexa Fluor® 488 conjugated CD44 and Alex Fluor® 647 conjugated SOX2 were purchased from Cell Signaling (Danvers, MA).

### Cell lines and xenograft

The EBV-positive NPC cell line, C666-1, was maintained in RPMI-1640 medium (Invitrogen, Carlsbad, CA) supplemented with 10% fetal bovine serum (FBS) (Invitrogen, Carlsbad, CA) and 1% penicillin and streptomycin (P/S) (Invitrogen, Carlsbad, CA)^3^. The EBV-negative NPC cell lines HONE1 and HK1 were maintained in DMEM medium supplemented with 5% FBS and 5% newborn calf serum (NCS) with 1% P/S^52^,^53^. Cells were cultured at 37°C in a 5% CO_2_ humidified incubator. The cell lines were authenticated by and obtained from the Hong Kong NPC AoE Cell Line Repository. The EBV-positive NPC xenograft, xeno-2117, was previously established from a NPC patient biopsy^54^ and was maintained subcutaneously in athymic BALB/c nu/nu mice for *in vivo* study.

### Cell growth assay

The cell growth assay was performed as previously described^55^. In brief, C666-1 cells (3x10^5^) were seeded onto 35mm culture dishes and treated with ICG-001 (10 µM). On day-3, 5, and 7 after ICG-001 treatment, the number of viable cells was determined and counted under inverted light microscope with trypan blue exclusion staining. In the cell growth assay of ICG-001 combined with cisplatin, C666-1 cells were treated with 1 µM ICG-001 and/or 1.5 µg/ml cisplatin. The number of viable cells was counted on day-7 after the drug treatment.

### Cell transfection

Transient transfection was performed using Lipofectamine Reagent 2000 (Invitrogen, Carlsbad, CA) according to the manufacturer's protocol. C666-1 cells (5x10^5^) were seeded onto fibronectin-coated 35-mm dishes. For siRNA knockdown, 50 nM ON-TARGETplus SMARTpool Human SOX2 siRNA (a pool of 4 siRNA: GCUCUUGGCUCCAUGGGUU; UCAUGAAGAAGGAUAAGUA; GCUUCUAGACCUACAUGAA; CAGUACAACUCCAUGACCA), or 50 nM ON-TARGETplus siCONTROL Non-Targeting siRNA (Dharmacon, Lafayette, CO) was transfected to C666-1. For miRNA precursor transfection, 50 nM pre-miR145 miRNA precursor or 50 nM pre-miR miRNA precursor control (Ambion, Austin, TX) was transfected to C666-1. After 72 hours of transfection, cells were harvested for expression analysis and tumor sphere formation assay.

### Luciferase reporter assay

C666-1 cells were transiently transfected with 50 ng pMirTarget luciferase reporter vector containing SOX2 3'UTR (Origene, Rockville, MD) along with 200 nM pre-miR145 miRNA precursor or pre-miR miRNA precursor control (Ambion, Austin, TX) using Lipofectamine Reagent 2000 (Invitrogen, Carlsbad, CA) mentioned above. The pMirTarget vector contains a red fluorescent protein (RFP) for monitoring the transfection efficiency and normalization. After 48 hours of transfection, the red fluorescent signal was captured under microscopy and then the cells were lysed for measurement using firefly luciferase reporter assay system (Promega, Valencia, CA) according to the manufacturer's manual. The luciferase activities were calculated and normalized to intensity of RFP expression.

### miRNA expression analysis

Total RNA was reverse-transcribed using TaqMan MicroRNA Assay Kit with miRNA-specific stem-looped RT primer (Applied Biosystems, Foster City, CA) according to the manufacturer's protocol. Realtime quantitative PCR was performed in Applied Biosystems StepOne Realtime PCR Systems using TaqMan Universal PCR Master Mix and TaqMan MicroRNA Assay primer (Applied Biosystems, Foster City, CA). Human U6 snRNA (RNU6B) was used as the endogenous control. The fold change in expression level was calculated using 2^-ΔΔCt^ method.

### Western blotting analysis

Western blotting analysis was performed as previously described^56^. In brief, cells were collected after 7 days of treatment, washed twice in phosphate-buffered saline (PBS), and lysed in ice-cold lysis buffer containing 1% phosphatase inhibitors cocktail (Calbiochem, San Diego, CA) and 0.25% protease inhibitors cocktail (Sigma, St. Louis, MO). Protein samples were resolved on SDS–polyacrylamide gel and transferred to PVDF membrane (Millipore, Billerica, MA). After blocking with 5% non-fat milk, membrane was incubated with primary antibodies (1:1000) and corresponding HRP-conjugated secondary antibodies (1:4000). Western-blotting substrate (Labfrontier Co. Ltd., Bio Division) was added to the membrane. The chemiluminescent signal was then detected and visualized on the X-ray film. β-actin or GAPDH was probed as an internal control.

### Tumor sphere formation assay and growth inhibition analysis on established tumor spheres

Tumor sphere formation assay was performed as previously described^55^. Cells were seeded in low cell density (2x10^3^ cells/well) on a 24-well ultra-low attachment plate (Corning, Acton, MA) in serum-free DMEM/F-12 (Invitrogen, Carlsbad, CA) supplemented with 20 ng/ml EGF (Sigma, St. Louis, MO), 20 ng/ml FGF (Cell Signaling, Danvers, MA), and 20 ng/ml IGF (Cell Signaling, Danvers, MA). The cultures were fed with fresh serum-free DMEM/F12 and growth factors every other day. ICG-001 (10 µM) was added to the culture on the first day of tumor sphere culturing. After 7 days of incubation, the images of cells were captured and tumor spheres having a diameter >20 μm were counted using Image J software. The total number and diameter of tumor spheres formed in ICG-001-treated and -untreated cultures were compared.

For studying the growth inhibitory effect of ICG-001 on established tumor spheres, tumor spheres were allowed to grow for 7 days without any drug treatment, ICG-001 (10 µM) was then added to the established tumor sphere cultures and the cells were incubated for a further 7 days. After incubation, the images of tumor spheres were captured under an inverted microscope for size profile and mean diameter analysis. Then the tumor spheres were harvested for immunofluorescence staining or fluorescence activated cell sorting (FACS) analysis.

### Immunofluorescence staining

The expression of SOX2 and CD44 was determined using immunofluorescence staining method as previously described^55^. Briefly, tumor spheres were collected, fixed, permeabilized, and incubated with Alex Fluor® 647 conjugated SOX2 and Alexa Fluor® 488 conjugated CD44 antibodies. The immunofluorescence images were captured using Olympus Fluoview 1000 confocal scanning laser microscope.

### FACS analysis

Quantitative analysis of SOX2 and CD44 expression by the spheroid cells was performed as previously described^55^. Briefly, the tumor spheres were collected and resuspended in PBS. The spheroid cells were fixed in 1.6 % paraformaldehyde, and permeabilized in ice-cold methanol. The cells were then washed and resuspended in 0.5% bovine serum albumin (BSA) in PBS, and stained with Alex Fluor® 647 conjugated SOX2 and Alexa Fluor® 488 conjugated CD44 antibodies. Respective mouse or rabbit IgG isotype controls were used as negative controls. For each sample, at least 10,000 cells were acquired and analyzed by FACSCalibur flow cytometer (Becton Dickinson, Franklin Lakes, NJ).

### MTT assay

MTT assay was performed as previously described^55^. In brief, C666-1 cells (1x10^4^) were seeded in triplicate in 96-well plate and incubated with various concentrations of ICG-001 and cisplatin. After 7 days of incubation, MTT solution (Sigma-Aldrich, St. Louis, MO) (0.25 mg/ml) was added and the optical density (OD) was measured at absorbance 540nm with reference to absorbance 690 nm. The OD is directly proportional to the number of proliferating cells and the percentage of cell proliferation was calculated compared to control wells.

### Analysis of combination index

The combination index (CI) was calculated using the Chou and Talalay method CI = (D)1/(Dx)1 + (D)2/(Dx)2 to determine the combined drug effects^57^. (D)1 and (D)2 are the doses of drug-1 and drug-2 used in combination that have x effect. (Dx)1 and (Dx)2 are the doses of drug-1 and drug-2 used alone that have x effect. The combined drug effect is considered as additive when CI = 1, synergistic when CI<1, and antagonistic when CI >1.

### Nude mouse tumorigenicity assay

For the C666-1 xenograft study, nude mice were supplied by the Laboratory Animal Unit of the University of Hong Kong and housed by Department of Clinical Oncology of Queen Elizabeth Hospital Hong Kong. All procedures were conducted under license from the Hong Kong Department of Health and approved by Committee on the Use of Live Animals in Teaching and Research (CULTAR) at the University of Hong Kong. For the xeno-2117 study, nude mice were supplied and housed by Department of Anatomical and Cellular Pathology and State Key Laboratory in Oncology in South China, The Chinese University of Hong Kong, under license from the Hong Kong Department of Health and approved by the Animal Experimentation Ethics Committee (AEEC) at The Chinese University of Hong Kong. In brief, 1x10^7^ C666-1 cells and an equal portion of xeno-2117 were subcutaneously (s.c.) injected into right flank of female athymic BALB/c nu/nu mice at 6-8 weeks of age, respectively. When the tumors reached 100mm^3^ for C666-1 and became palpable for xeno-2117, respectively, the mice were divided into four groups (n = 4) for C666-1 xenograft and five (n = 5) for xeno-2117 for subsequent treatment either with drug vehicle control, ICG-001 alone (50 mg/kg/day dissolved in PBS), cisplatin alone (3.5 mg/kg/week dissolved in 3.3% DMF in saline for C666-1 xenografts and 3 mg/kg/week dissolved in 3.3% DMF in saline for xeno-2117), or combined ICG-001 (50 mg/kg/day dissolved in PBS) with cisplatin (3.5 mg/kg/week dissolved in 3.3% DMF in saline for C666-1 xenografts and 3 mg/kg/week dissolved in 3.3% DMF in saline for xeno-2117). For stable and continuous delivery of ICG-001, osmotic minipumps Alzet Model 1004 (DURECT Corporation, Cupertino, CA) were s.c. implanted to the left flanks of the ICG-001 treatment and combined treatment group of mice according to manufacturer's instructions. Osmotic minipumps containing PBS as ICG-001 solvent control were s.c. implanted to vehicle control group and cisplatin treatment group. The day of osmotic minipump implantation was referred as day 0. Three days post-tansplantation, cisplatin was administrated by intraperitoneal (i.p.) injection to cisplatin treatment group and combined treatment group weekly. For vehicle control group and ICG-001 treatment group, 3.3% DMF in saline was administrated by i.p. injection weekly as cisplatin solvent control. The tumor volume in mm^3^ (length x width x height) and the mice body weight were measured twice weekly, until tumor volume in control group became too large for the mouse.

### Statistical analysis

All results were representative results from at least three independent experiments. Error bars in each data point represented the arithmetic mean ± SD of three replicates (n = 3). The *p*-values were calculated by Student's *t*-test, *p* < 0.05 was considered as statistically significant.

## Author Contributions

K.C.C., T.T.C.Y., R.K.C.N., R.N.S.W., K.W.L., W.T.N, A.W.M.L., G.S.W.T., M.K., M.L.L. and N.K.M. involved in project design. K.C.C. and N.K.M. wrote the manuscript. M.K. and M.L.L edited the manuscript. K.C.C. and L.S.C. performed the *in vitro* experiments. K.C.C., L.S.C., C.L., and J.C.Y.I. performed nude mouse experiment. K.C.C. and L.S.C. performed data analysis. All authors reviewed the manuscript.

## Supplementary Material

Supplementary Information Supplementary Information
