# Supplementary material for: Therapeutic targeting of CBP/β-catenin signaling reduces cancer stem-like population and synergistically suppresses growth of EBV-positive nasopharyngeal carcinoma cells with cisplatin
Source: Sci Rep. 2015 Apr 21;5:9979. doi: 10.1038/srep09979 (PMC4404684; doi:10.1038/srep09979)
Supplement: Supplementary Information [file srep09979-s1.pdf]

Therapeutic targeting of CBP/β-catenin signaling reduces cancer stem-like population and synergistically suppresses growth of EBV-positive nasopharyngeal carcinoma cells with cisplatin

King Chi Chan[1], Lai Sheung Chan[1], Joseph Chok Yan Ip[2], Carman Lo[3], Timothy Tak Chun Yip[4,5], Roger Kai Cheong Ngan[4,5], Ricky Ngok Shun Wong[1,5], Kwok Wai Lo[3], Wai Tong Ng[5,6], Anne Wing Mui Lee[5,6], George Sai Wah Tsao[5,7], Michael Kahn[8], Maria Li Lung[2,5], and Nai Ki Mak[1,5,*]

[1]Department of Biology, Hong Kong Baptist University, P.R. China

[2]Department of Clinical Oncology, University of Hong Kong, P.R. China

[3]Department of Anatomical and Cellular Pathology and State Key Laboratory in Oncology in South China, The Chinese University of Hong Kong, P.R. China

[4]Department of Clinical Oncology, Queen Elizabeth Hospital Hong Kong, P.R. China

[5]Center for Nasopharyngeal Carcinoma Research, University of Hong Kong, P.R. China

[6]Clinical Oncology, Pamela Youde Nethersole Eastern Hospital, P.R. China

[7]Department of Anatomy, University of Hong Kong, P.R. China

[8]Department of Biochemistry and Molecular Biology, Norris Comprehensive Cancer Center, Keck School of Medicine, University of Southern California, Los Angeles, CA, USA

***Corresponding author at:** Department of Biology, Hong Kong Baptist University, P.R. China. Tel.: +852 3411-7059; E-mail: nkmak@hkbu.edu.hk

Supplementary information

Full-length blots:
Figure S1

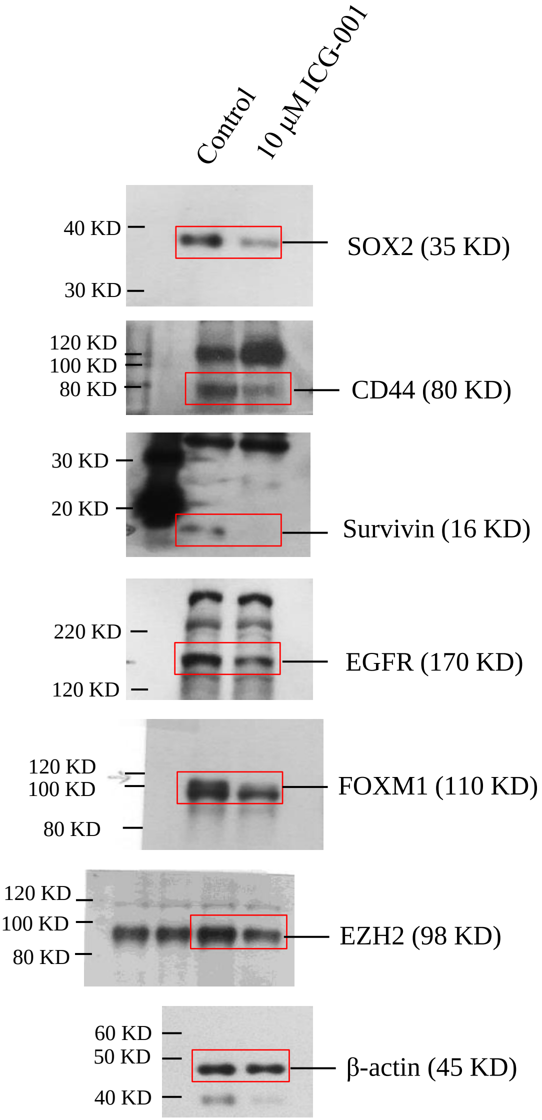

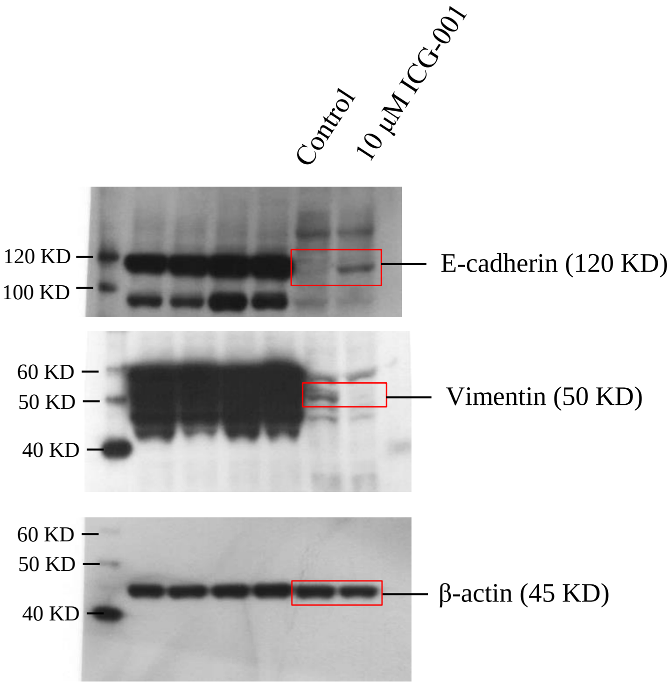

Figure S2

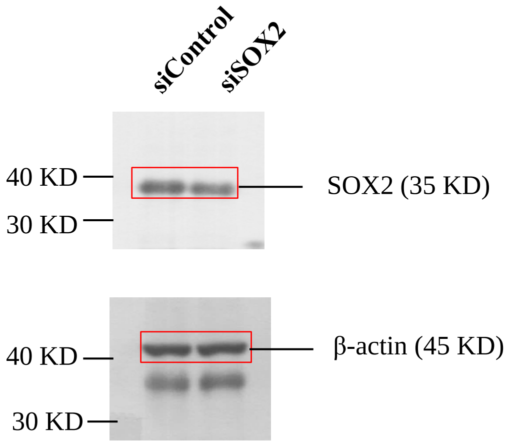

Figure S3

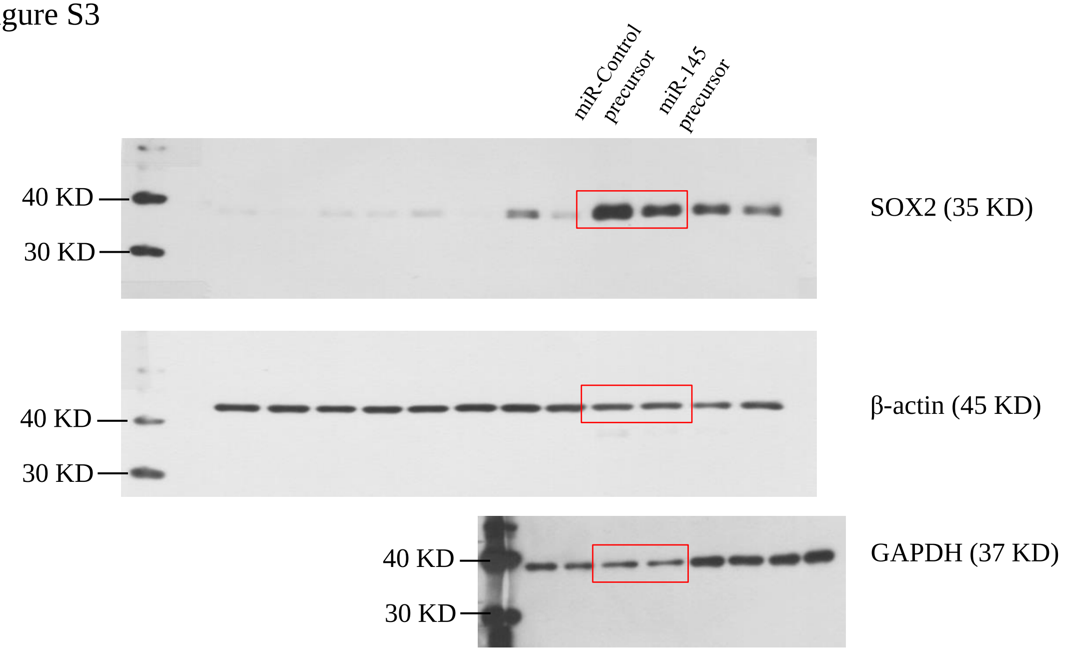

miR-Control precursor
miR-145 precursor
40 KD
30 KD
SOX2 (35 KD)
40 KD
30 KD
β-actin (45 KD)
40 KD
30 KD
GAPDH (37 KD)
